# Neuroprotective Effects of Ischemic Preconditioning and Postconditioning on Global Brain Ischemia in Rats through the Same Effect on Inhibition of Apoptosis

**DOI:** 10.3390/ijms13056089

**Published:** 2012-05-18

**Authors:** Zhe-Min Ding, Bing Wu, Wei-Qiao Zhang, Xiao-Jie Lu, Yu-Chang Lin, Yong-Jian Geng, Yi-Feng Miao

**Affiliations:** 1 Neuroscience Center, Wuxi No. 2 Hospital, Nanjing Medical University, Wuxi 214002, China; E-Mails: godlovesatan@163.com (Z.-M.D.); xiaojieluwuxi@163.com (X.-J.L.); linyuchang@sina.com (Y.-C.L.); 2 Department of Neurosurgery, Ren Ji Hospital, Shanghai Jiao Tong University School of Medicine, Shanghai 200127, China; E-Mail: wbnmu@hotmail.com; 3 Department of Neurosurgery, Yuyao People’s Hospital, Yuyao 315472, China; E-Mail: zhang951154@sina.com; 4 University of Texas Medical School at Houston, Houston, TX 77030, USA; E-Mail: yong-jian.geng@uth.tmc.edu

**Keywords:** brain ischemic injury, ischemic preconditioning, ischemic postconditioning, apoptosis, neuroprotection

## Abstract

Transient forebrain or global ischemia induces neuronal death in vulnerable CA1 pyramidal cells with many features. A brief period of ischemia, *i.e.*, ischemic preconditioning, or a modified reperfusion such as ischemic postconditioning, can afford robust protection of CA1 neurons against ischemic challenge. Therefore, we investigated the effect of ischemic preconditioning and postconditioning on neural cell apoptosis in rats. The result showed that both ischemic preconditioning and postconditioning may attenuate the neural cell death and DNA fragment in the hippocampal CA1 region. Further western blot study suggested that ischemic preconditioning and postconditioning down-regulates the protein of cleaved caspase-3, caspase-6, caspase-9 and Bax, but up-regulates the protein Bcl-2. These findings suggest that ischemic preconditioning and postconditioning have a neuroprotective role on global brain ischemia in rats through the same effect on inhibition of apoptosis.

## 1. Introduction

Brain ischemia is becoming a leading cause of morbidity and mortality world-wide [1]. Its major pathophysiological manifestation is acute brain ischemia-reperfusion injury [2].

A variety of clinical trials of pharmacological neuroprotective strategies in stroke have been disappointing [3–5]. Therefore, innovative treatment strategies for protecting the brain against the detrimental effects of this form of injury are required in order to improve clinical outcomes in patients with brain ischemic injury. Researchers are now interested in the brain’s endogenous strategies for neuroprotection [6,7]. Harnessing the endogenous protection elicited by the brain’s ability to “condition” itself has recently emerged as a powerful new strategy for limiting brain injury [8,9]. The concept of ischemic preconditioning is defined as a brief noninjurious episode of ischemia which can protect the brain from a subsequent longer ischemic insult [10,11]. This strategy to attenuate the pathophysiological consequences of ischemic injury would be focused on pretreatment situations, such as protection before bypass surgery. But a non-pharmacological neuroprotective strategy for therapeutic application after ischemia onset remains elusive. Recently, many researchers demonstrated that a modified reperfusion in the same artery occluded to produce a prolonged episode of focal cerebral ischemia may confer postischemic neuroprotection [12]. This phenomenon was termed ischemic postconditioning.

Some studies have suggested that pre- and postconditioning are similar phenomena with the effectors being a similar group of downstream signaling cascades [13]. Other studies suggest that the mechanisms regulating postconditioning may be entirely different from preconditioning [14]. The reason is that the rapidity of onset of postconditioning-induced neuroprotection contrasts with a significant temporal delay for protein synthesis-dependent, preconditioning-induced neuroprotection.

Therefore, we investigated the neuroprotective effect of ischemic preconditioning and postconditioning on global brain ischemia in rats. We further identified the effect of ischemic preconditioning and postconditioning on neural cell apoptosis and apoptosis-related proteins. Our results showed that ischemic preconditioning and postconditioning have a relatively equal neuroprotective role on global brain ischemia in rats through the same effect on inhibition of neuronal apoptosis.

## 2. Results and Discussion

### 2.1. Neuronal Injury Following Global Cerebral Ischemia Is Reduced by Ischemic Preconditioning and Postconditioning

**Figure 1:**
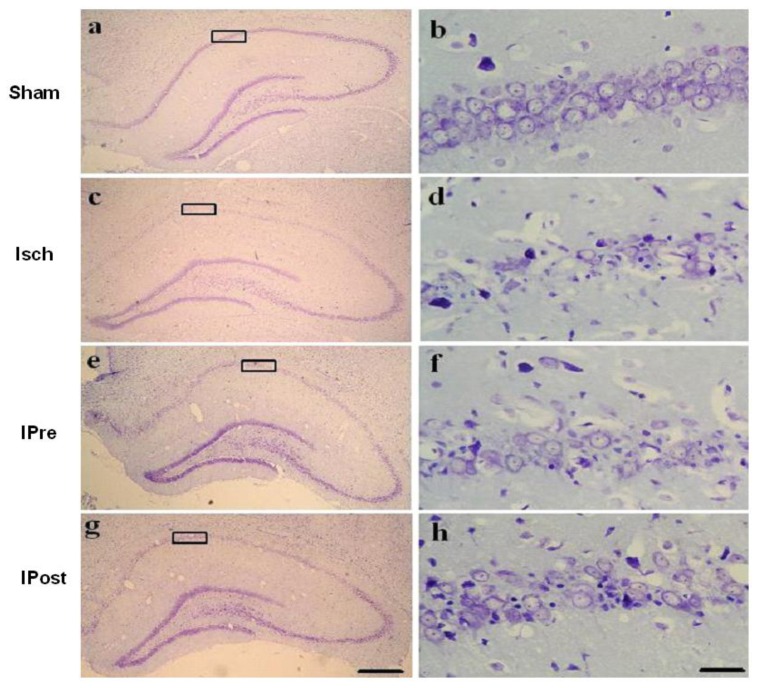

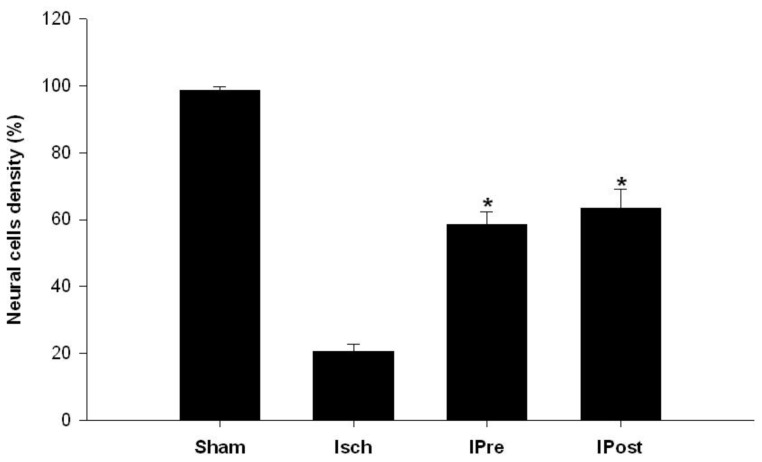
Effects of ischemic preconditioning and postconditioning on ischemia-induced neuronal cell loss in hippocampal CA1 regions. Animals were subjected to sham operation (Sham, **a**, **b**), global ischemia (Isch, **c**, **d**), preconditioning (IPre, **e**, **f**) and postconditioning (IPost, **g**, **h**). Three days later, brains were fixed with 4% paraformaldehyde followed by preparation of coronal sections from frozen brains and subsequent staining with cresyl violet to determine cell survival in neuronal layers of the hippocampi (*n* = 5). The boxed areas of CA1 subfield are shown at higher magnification, and the number of viable neurons in these areas was counted. Images of hippocampi at lower magnification (×40) are **a**, **c**, **e** and **g**, and images at higher (×400) are **b**, **d**, **f**, and **h**. Scale bar = 30 μm. Data are mean ± S.D. ^*^ *p* < 0.01 by nonparametric ANOVA followed by Dunn’s analysis comparing with ischemic rats.

We examined whether preconditioning and postconditioning were associated with an increase in neuronal cell survival in the hippocampal CA1 region after ischemia. For rats in the sham group, the CA1 region contained 3 to 4 layers of pyramidal cells in a regular arrangement under cresyl violet staining (Figure 1a). At high magnification, the pyramidal cells were large, round, transparent and had intact nuclei (Figure 1b). In contrast, three days after lethal ischemia, most CA1 pyramidal cells were shrunken with pyknotic nuclei (Figure 1c,d). In the case of an ischemic insult, neuronal density was significantly increased by preconditioning and postconditioning compared to the ischemic insult alone (*p* < 0.01, Figure 1c–h). Hippocampal CA1 cell counts revealed that the percentages of viable neurons in the Isch, IPre, and IPost groups were 20.7 ± 2.1%, 58.6 ± 3.8% and 63.5 ± 5.6%, respectively (Figure 1, *p* < 0.01).

**Figure 2:**
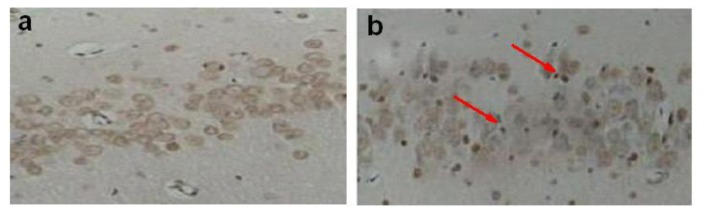

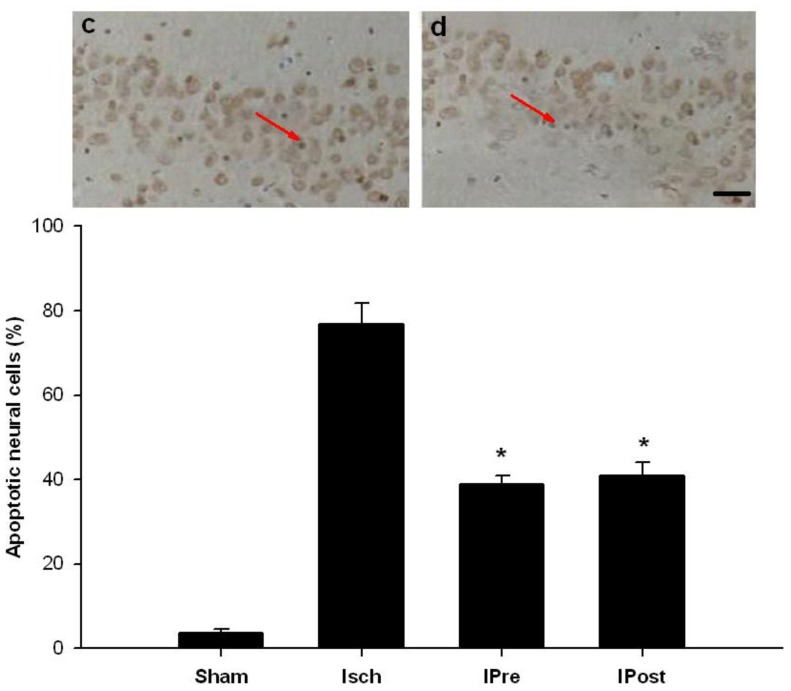
Effects of ischemic preconditioning and postconditioning on ischemia-induced neuronal apoptosis in hippocampal CA1 regions. Animals were subjected to sham operation (Sham, **a**), global ischemia (Isch, **b**), preconditioning (IPre, **c**) and postconditioning (IPost, **d**). Three days later, animals were killed and coronal sections (18 mm) of fresh-frozen rat brain were cut by cryotome. Sections were fixed in 4% paraformaldehyde for 60 min at room temperature and processed for TUNEL nuclear staining (*n* = 5). Scale bar = 30 μm. The arrows point to the apoptotic cells. Data are mean ± S.D. ^*^ *p* < 0.01 by nonparametric ANOVA followed by Dunn’s analysis comparing with ischemic rats.

TUNEL staining was applied to examine DNA fragmentation in neurons undergoing apoptosis after ischemia. TUNEL labeling was undetectable in the CA1 region in sham groups (Figure 2a). But global ischemia induced a marked increase in the incidence of TUNEL-positive CA1 neurons (Figure 2b). Preconditioning and postconditioning significantly blocked ischemia-induced DNA fragmentation indicated by TUNEL (*p* < 0.01, Figure 2c,d). The number of apoptotic cells in the CA1 region in the Isch, IPre, and IPost groups were 76.8 ± 5.1%, 38.9 ± 2.1% and 40.9 ± 3.2%, respectively (Figure 2, *p* < 0.01).

### 2.2. Downregulation of Hippocampal Cleaved Caspase-3, Cleaved Caspase-6, Cleaved Caspase-9 and Bax

**Figure 3:**
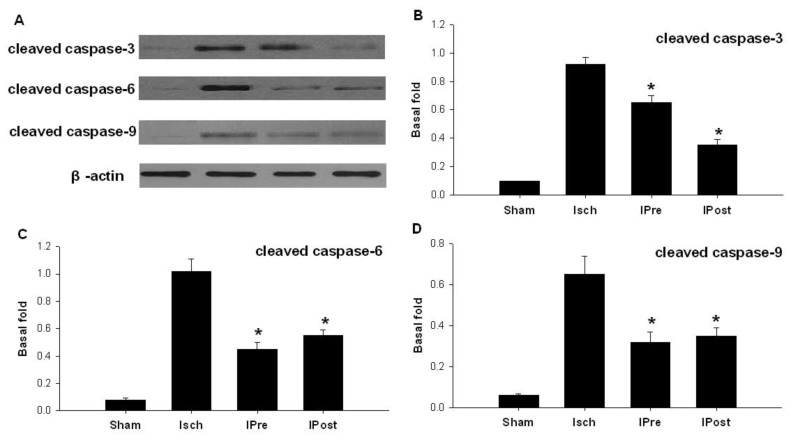
Western blotting analysis of cleaved caspase expressions in hippocampal CA1 regions. Animals were subjected to sham operation (Sham), global ischemia (Isch), preconditioning (IPre) and postconditioning (IPost). Three days later, extracts from the hippocampi of the rats and sham controls were subjected to western blotting with anti-cleaved caspase-3 (**B**), anti-cleaved caspase-6 (**C**) and anti-cleaved caspase-9 (**D**) protein. Data are mean ± S.D. (*n* = 5). ^*^ *p* < 0.01 by nonparametric ANOVA, followed by Dunn’s analysis comparing with ischemic rats.

**Figure 4:**
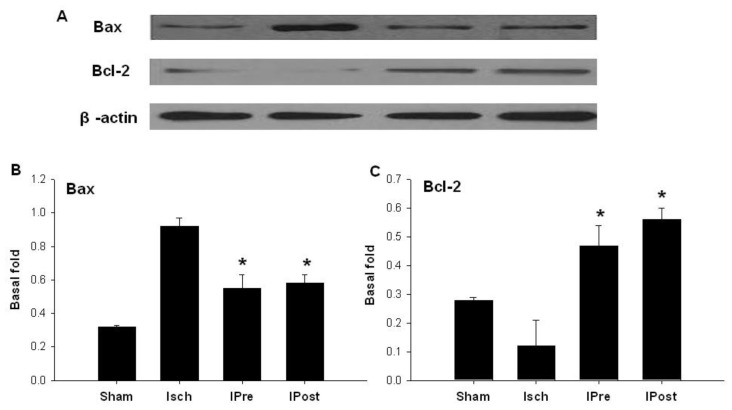
Western blotting analysis of Bax and Bcl-2 expressions in hippocampal CA1 regions. Animals were subjected to sham operation (Sham), global ischemia (Isch), preconditioning (IPre) and postconditioning (IPost). Three days later, extracts from the hippocampi of the rats and sham controls were subjected to western blotting with anti-Bax (**B**) and anti-Bcl-2 (**C**) protein. Data are mean ± S.D. (*n* = 5). ^*^ *p* < 0.01 by nonparametric ANOVA, followed by Dunn’s analysis comparing with ischemic rats.

Western blot was used to assess the effect of global ischemia, ischemic preconditioning and postconditioning on apoptosis-related protein expression. Western blot analysis revealed that three days after reperfusion, the expression levels of cleaved caspase-3 in the sham, Isch, IPre, and IPost groups were 0.11 ± 0.04, 0.92 ± 0.05, 0.65 ± 0.05 and 0.35 ± 0.04, respectively (Figure 3a,b). While the expression levels of cleaved caspase-6 in the sham, Isch, IPre, and IPost groups were 0.08 ± 0.01, 1.02 ± 0.09, 0.45 ± 0.05 and 0.55 ± 0.04, respectively (Figure 3a,c). The expression levels of cleaved caspase-9 in the sham, Isch, IPre, and IPost groups were 0.06 ± 0.01, 0.65 ± 0.09, 0.32 ± 0.06 and 0.35 ± 0.05, respectively (Figure 3a,d). Also, our data found that the expression levels of Bax in the sham, Isch, IPre, and IPost groups were 0.32 ± 0.01, 0.92 ± 0.05, 0.55 ± 0.08 and 0.58 ± 0.05, respectively (Figure 4a,b, *p* < 0.01). The results showed that both ischemic preconditioning and postconditioning downregulate the expression of cleaved caspase-3, caspase-6, caspase-9 and Bax (Figures 3,4, *p* < 0.01).

### 2.3. Upregulation of Hippocampal Bcl-2

We detected the expression of Bcl-2 in the CA1. Western blot analysis showed that three days after reperfusion, the expression levels of Bcl-2 in the sham, Isch, IPre, and IPost groups were 0.28 ± 0.01, 0.12 ± 0.09, 0.47 ± 0.07 and 0.56 ± 0.04, respectively (Figure 4a,c, *p* < 0.01). The results indicated that ischemic preconditioning as well as ischemic postconditioning could upregulate the expression of Bcl-2 in the CA1 after ischemic insult in rats (Figure 4, *p* < 0.01).

### 2.4. Discussion

Our studies showed that lethal ischemic insult for 10 min caused abundant neuronal cell death and apoptosis in the hippocampal CA1 region. However, when the animals were pretreated with preconditioning for 3 min, two days before the lethal ischemic insult, the above neuronal injury was significantly attenuated. At the same time, after the modified reperfusion, in which rats were subjected to six cycles of 10 s reperfusion and 10 s ischemia, the ischemic injury was also markedly reduced. We further found that both ischemic preconditioning and postconditioning could down-regulate the expression of cleaved caspase-3, cleaved caspase-6, cleaved caspase-9 and Bax, but up-regulate the protein Bcl-2 in the hippocampus. This suggests that preconditioning and postconditioning have the same effect on anti-apoptosis against the ischemia-reperfusion injury.

Brain ischemic injury, resulting either from global or focal decreases in perfusion, is among the most common and important causes of disability and death worldwide [1]. These patients may experience a variety of difficulties such as dyskinesia, sensory disturbance, memory impairment, and many emotional and social problems following the ischemic injury [15]. However, until now, most of the symptoms could not be improved by psychological or psychiatric treatment [16,17]. Neuroprotection or brain repair in patients after acute brain ischemic injury is a major unmet medical need. Pharmacological treatments are either ineffective or confounded by adverse effects [18]. Consequently, endogenous mechanisms by which the brain protects itself against noxious stimuli and recovers from damage are being studied [19].

Ischemic conditioning offers a way to induce endogenous neuroprotection [20]. Ischemic conditioning has been defined as the practice of applying brief episodes of nonlethal ischemia and reperfusion to confer protection against a sustained episode of lethal ischemia and reperfusion injury. Now, it is regarded as one potential therapeutic strategy. Importantly, the protective stimulus can be applied before (ischemic preconditioning) or after (ischemic postconditioning) onset of the sustained episode of lethal ischemia. But until now, the basic biology of both preconditioning and postconditioning has been unclear [21]. The major unknowns for both conditioning responses include the process or substances that trigger the cascade of events which will lead to attenuation of reperfusion injury. In our previous studies [11,22], we have investigated the effect of ischemic preconditioning or ischemic postconditioning. Our studies have demonstrated that both ischemic preconditioning and postconditioning may protect neuron injury after brain ischemic brain. We ask if they have the same neuroprotective mechanism which may become the same brain protective target. Therefore, in our study we investigated if both pre- and postconditioning can trigger anti-apoptotic function.

The term “programmed cell death” (apoptosis) is characterized by a series of well-defined and distinct morphological and biochemical changes [23]. Apoptosis is triggered following cerebral ischemia by a variety of death signals, especially caspase activation [24,25]. Caspases, or cysteine-dependent, aspartate-directed proteases, are a family of cysteine proteases that play essential roles in apoptosis. Scientists have termed caspases as “executioner” proteins. There are two types of apoptotic caspases: initiator (apical) caspases and effector (executioner) caspases. Caspase-9 belongs to the initiator caspases, but the caspase-3 and caspase-6 are related to effector caspases. So, in this study, we detected the protein level of caspase-3, -6 and -9. Our results showed that the postconditioning can down-regulate the protein more than the pre-conditioning. Although there are no significant differences on the caspase-6 and caspase-9 level, the level of caspase-6 and caspase-9 in the pre-conditioning group is higher than postconditioning. This suggests a balance among the caspase family.

The Bcl-2 family proteins are located at outer mitochondria membranes, and it controls the activation of downstream caspases, thus representing a critical proximal intracellular checkpoint in the mitochondria-mediated apoptosis pathway [26,27]. Therefore, western blotting was used to detect the protein of caspases and Bcl-2. The results of this study showed that both ischemic preconditioning and postconditioning downregulate the expression of hippocampal cleaved caspase-3, caspase-6, caspase-9 and Bax, but upregulate hippocampal Bcl-2 expression. We found that both ischemic preconditioning and postconditioning have the anti-apoptotic function through the same mechanism.

Until now, there are a number of mechanisms [28–30] which may be related to the conditioning effect on ischemic injury, especially in coronary heart disease. A variety of key molecules and signaling pathways has been found, including GABA receptor, iNOS, adenosine, heat shock protein. Also, many novel conditioning methods have been found, such as remote ischemic conditioning [28]. It is certain that ischemic tolerance can protect neurons, and at the same time, it also can maintain brain function. But there has been little study about the relationship between such kinds of conditioning, or cross-tolerance. Our further study will focus on the common mechanisms about all kinds of conditioning methods which may allow us to exploit the protective effect pharmacologically.

## 3. Experimental Section

### 3.1. Animals

Age-matched adult male Sprague-Dawley rats weighing 200–220 g (Nanjing Medical University Animal Laboratories) were maintained in a temperature- and light-controlled environment with a 14/10-h light/dark cycle. All subjects were treated in accordance with the principles and procedures of the Animal Care and Experimental Committee of the Nanjing Medical University.

### 3.2. Establishment of the 4-Vessel Occlusion Model

Global ischemia was induced using the 4-vessel occlusion method, as described previously [31]. Briefly, rats were anesthetized by intraperitoneal administration of 20% chloral hydrate (350 mg/kg) on day 1. Both vertebral arteries were electrocauterized, and the bilateral common carotid arteries were separated. On day 2, the bilateral common carotid arteries of conscious rats were occluded with aneurysm clips for 10 min. During surgery, body temperature was monitored and maintained at 37.5 ± 0.5 °C with a rectal thermistor and a heat lamp. Rats that had lost the righting reflex, had dilated pupils and were unresponsive to light were used in experiments.

### 3.3. Ischemic Preconditioning and Postconditioning

**Figure 5:**
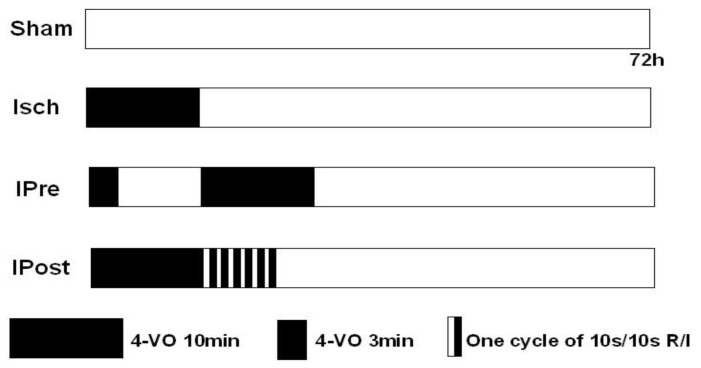
Experimental protocol used to evaluate the effect of ischemic preconditioning and postconditioning after ischemia-reperfusion. Sham: sham-operated rats; Isch: rats subjected to 10 min of 4-vessel occlusion (VO) followed by reperfusion; IPre: rats subjected to a three-minute occlusion that took place 48 h prior to the global ischemic event; IPost: rats subjected to 10 min of 4-vessel occlusion followed by postconditioning. Postconditioning reperfusion was performed for 10 s, then the bilateral common carotid arteries were re-occluded for 10 s and this procedure was repeated 6 times.

Rats were randomly assigned to four groups (Figure 5). For the ischemic preconditioning group (IPre), preconditioning consisted of a three-minute occlusion that took place 48 h prior to the global ischemic event. For the ischemic postconditioning group (IPost), global ischemia was induced by occluding the common arteries with aneurysm clips, and reperfusion was performed by removing the clips. Immediately after reperfusion, rats in the IP group were subjected to six cycles of 10 s reperfusion and 10 s ischemia. In the sham group (Sham), rats were subjected to the same anesthesia and surgical procedures, except that the carotid arteries were not occluded. The protocol is shown in Figure 5.

### 3.4. Histological Analysis

Neuronal cell loss was assessed by histological examination of the dorsal hippocampus (CA1 region) of brain sections stained with cresyl violet. Animals were sacrificed 72 h after the sham, Isch, IPre and IPost surgeries. Animals were deeply anesthetized with halothane and fixed by transcardiac perfusion with ice-cold 4% paraformaldehyde in PBS (0.1 M, pH 7.4). Brains were removed and immersed in fixative. Coronal sections (15 μm) were cut at the level of the dorsal hippocampus with a cryotome and stained with cresyl violet. The number of live pyramidal neurons per 250-mm length of the medial CA1 region was counted under a light microscope at ×40 magnification. Four sections were counted from each rat, with each experimental group consisting of five rats.

### 3.5. *In Situ* Labeling of DNA Fragmentation by TUNEL

To detect DNA fragmentation in degenerating neurons, animals were sacrificed 72 h after reperfusion, and coronal sections (18 mm) of freshly-frozen rat brain were cut using a cryotome. Sections were fixed in 4% paraformaldehyde for 60 min at room temperature and processed for TUNEL nuclear staining using an *in situ* cell death detection kit (Roche Molecular Biochemicals and Molecular Probes; manufacturer’s instructions were followed). Images were viewed under a Nikon ECLIPSE TE300 fluorescence microscope and acquired with a SPOT RT CCD-cooled camera equipped with diagnostic software version 3.0. Imaging settings were kept constant across experimental and control groups. TUNEL-positive cells were identified directly by the fluorescence signal of incorporated fluorescein-dUTP. Cells in 32 fields sampled from the CA1 region of five animals were scored.

### 3.6. Western Blotting

Tissue samples were lysed in RIPA lysis buffer on ice for 30 min and centrifuged at 16,000 revolutions per minute for 30 min at 4 °C. Protein concentration was determined using the bicinchoninic acid method. Loading buffer was added to each sample (60 μg) and samples were boiled for 5 min. Samples were run on sodium dodecyl sulfate polyacrylamide gels and then transferred to polyvinylidene fluoride membranes (Immobilon, Millipore, Billerica, MA, USA). The membranes were blocked with 5% milk in Tris-buffered saline with Tween (TBST) for 1 h and incubated with the following primary antibodies at 4 °C overnight: rabbit anti-cleaved caspase-3, anti-cleaved caspase-6, anti-cleaved caspase-9, anti-Bax and anti-Bcl-2 (1:2000; Abcam, Cambridge, MA, USA) and mouse anti-β-actin (1:1000; Sigma-Aldrich, St. Louis, MO, USA). The membranes were washed and incubated with the following secondary antibodies for 1 h at room temperature in the dark: goat anti-rabbit IRDye 800 CW (1:4000; Kirkegaard and Perry Laboratories [KPL], Gaithersburg, MD, USA) and goat anti-mouse IRDye 800 CW (1:4000; Li-COR Biosciences, Lincoln, NE, USA). Then the membranes were scanned with an Odyssey Infrared Imaging System (Li-COR Biosciences) for analysis of the relative level of apoptosis-related protein expression. The protein gray value was divided by the β-actin gray value.

### 3.7. Statistical Analysis

All data are presented as the mean ± S.E.M. The statistical significance was examined by ANOVA, followed by Dunn's test. Differences were considered significant at *p* < 0.05. The statistical software SPSS 13.0 (SPSS Inc, Chicago, IL) was used for the statistical analyses.

## 4. Conclusions

In conclusion, the results of this study showed that both ischemic preconditioning and postconditioning can protect the ischemic brain injury in rats through the same effect on inhibition of apoptosis. Further study will emphasize how to induce an optimal algorithm of combination of pre- and post-neuroprotective effects.

## References

[1] Wardlaw J.M., von Kummer R., Farrall A.J., Chappell F.M., Hill M., Perry D. (2010). A large web-based observer reliability study of early ischaemic signs on computed tomography. The Acute Cerebral CT Evaluation of Stroke Study (ACCESS). PLoS One.

[2] Sinanovic O. (2010). Neuropsychology of acute stroke. Psychiatr. Danubina.

[3] Van Bel F., Groenendaal F. (2008). Long-term pharmacologic neuroprotection after birth asphyxia: Where do we stand?. Neonatology.

[4] Mantz J., Degos V., Laigle C. (2010). Recent advances in pharmacologic neuroprotection. Eur. J. Anaesth.

[5] Chavez J.C., Hurko O., Barone F.C., Feuerstein G.Z. (2009). Pharmacologic interventions for stroke: Looking beyond the thrombolysis time window into the penumbra with biomarkers, not a stopwatch. Stroke J. Cereb. Circ.

[6] Durukan A., Tatlisumak T. (2010). Preconditioning-induced ischemic tolerance: A window into endogenous gearing for cerebroprotection. Exp. Transl. Stroke Med.

[7] Bhuiyan M.I.H., Kim Y.J. (2010). Mechanisms and prospects of ischemic tolerance induced by cerebral preconditioning. Int. Neurourol. J.

[8] Lehotsky J., Burda J., Danielisova V., Gottlieb M., Kaplan P., Saniova B. (2009). Ischemic tolerance: The mechanisms of neuroprotective strategy. Anat. Rec. (Hoboken).

[9] Ferrara A., El Bejaoui S., Seyen S., Tirelli E., Plumier J.-C. (2009). The usefulness of operant conditioning procedures to assess long-lasting deficits following transient focal ischemia in mice. Behav. Brain Res.

[10] Stenzel-Poore M.P., Stevens S.L., King J.S., Simon R.P. (2007). Preconditioning reprograms the response to ischemic injury and primes the emergence of unique endogenous neuroprotective phenotypes: A speculative synthesis. Stroke J.Cereb. Circ.

[11] Miao Y., Zhang W., Lin Y., Lu X., Qiu Y. (2010). Neuroprotective effects of ischemic preconditioning on global brain ischemia through up-regulation of acid-sensing ion channel 2a. Int. J. Mol. Sci.

[12] Xing B., Chen H., Zhang M., Zhao D., Jiang R., Liu X., Zhang S. (2008). Ischemic postconditioning inhibits apoptosis after focal cerebral ischemia/reperfusion injury in the rat. Stroke J. Cereb. Circ.

[13] Hausenloy D.J., Yellon D.M. (2009). Preconditioning and postconditioning: Underlying mechanisms and clinical application. Atherosclerosis.

[14] Pignataro G., Scorziello A., di Renzo G., Annunziato L. (2009). Post-ischemic brain damage: Effect of ischemic preconditioning and postconditioning and identification of potential candidates for stroke therapy. FEBS J.

[15] Durukan A., Tatlisumak T. (2007). Acute ischemic stroke: Overview of major experimental rodent models, pathophysiology, and therapy of focal cerebral ischemia. Pharmacol. Biochem. Behav.

[16] Plum F. (2001). Neuroprotection in acute ischemic stroke. J. Am. Med. Assoc.

[17] Ginsberg M.D. (2009). Current status of neuroprotection for cerebral ischemia. Synoptic overview. Stroke.

[18] Gladstone D.J., Black S.E., Hakim A.M. (2002). Toward wisdom from failure-Lessons from neuroprotective stroke trials and new therapeutic directions. Stroke.

[19] Kirino T. (2002). Ischemic tolerance. J. Cereb. Blood Flow Metab.

[20] Hausenloy D.J., Yellon D.M. (2011). The therapeutic potential of ischemic conditioning: An update. Nat. Rev. Cardiol.

[21] Kardesoglu E., Isilak Z., Uz O., Yiginer O. (2011). Ischemic conditioning: A current concept in reducing reperfusion injury. Chin. Med. J.

[22] Zhang W., Miao Y., Zhou S., Jiang J., Luo Q., Qiu Y. (2011). Neuroprotective effects of ischemic postconditioning on global brain ischemia in rats through upregulation of hippocampal glutamine synthetase. J. Clin. Neurosci.

[23] Kocher A.A., Schuster M.D., Szabolcs M.J., Takuma S., Burkhoff D., Wang J., Homma S., Edwards N.M., Itescu S. (2001). Neovascularization of ischemic myocardium by human bone-marrow-derived angioblasts prevents cardiomyocyte apoptosis, reduces remodeling and improves cardiac function. Nat. Med.

[24] Vohra H.A., Galinanes M. (2005). Effect of the degree of ischaemic injury and reoxygenation time on the type of myocardial cell death in man: Role of caspases. BMC Physiol.

[25] Prunell G.F., Arboleda V.A., Troy C.M. (2005). Caspase function in neuronal death: Delineation of the role of caspases in ischemia. CNS Neurol. Disord. Curr. Drug Target.

[26] Yang X.-Y., Liu Q.-N., Zhang L., Jiang S.-Q., Gong P.-L. (2010). Neuroprotective effect of dauricine after transient middle cerebral artery occlusion in rats: Involvement of Bcl-2 family proteins. Am. J. Chin. Med.

[27] Koubi D., Jiang H., Zhang L., Tang W., Kuo J., Rodriguez A.I., Hunter T.J., Seidman M.D., Corcoran G.B., Levine R.A. (2005). Role of Bcl-2 family of proteins in mediating apoptotic death of PC12 cells exposed to oxygen and glucose deprivation. Neurochem. Int.

[28] Lim S.Y., Hausenloy D.J. (2012). Remote ischemic conditioning: From bench to bedside. Front. Physiol.

[29] Thielmann M. (2012). Remote ischemic preconditioning in cardiac surgery: Caught between clinical relevance and statistical significance?. Basic Res. Cardiol.

[30] Yagi T., Yoshioka H., Wakai T., Kato T., Horikoshi T., Kinouchi H. (2011). Activation of signal transducers and activators of transcription 3 in the hippocampal CA1 region in a rat model of global cerebral ischemic preconditioning. Brain Res.

[31] Hossmann K.A. (2008). Cerebral ischemia: Models, methods and outcomes. Neuropharmacology.

